# Incidence, Diagnoses, and Outcomes of Pediatric Nontraumatic Chest Pain Attended by Ambulance

**DOI:** 10.1001/jamanetworkopen.2025.33962

**Published:** 2025-09-26

**Authors:** Daniel Okyere, Emily Nehme, Emily Mahony, Dion Stub, Luke P. Dawson, Jocasta Ball, Ben Meadley, David Anderson, Tegwyn McManamny, Ziad Nehme

**Affiliations:** 1 Centre for Research and Evaluation, Ambulance Victoria, Blackburn North, Victoria, Australia; 2 School of Public Health and Preventive Medicine, Monash University, Prahran, Victoria, Australia; 3 Department of Paramedicine, Monash University, Frankston, Victoria, Australia; 4 Department of Cardiology, The Alfred Hospital, Melbourne, Victoria, Australia; 5 Monash Alfred Baker Centre for Cardiovascular Research, Monash University, Prahran, Victoria, Australia

## Abstract

**Importance:**

Nontraumatic chest pain is a common clinical presentation, but less is known about it in children seeking emergency medical services (EMS) care.

**Objective:**

To determine the incidence, diagnoses, and outcomes of pediatric EMS-attended nontraumatic chest pain.

**Design, Setting, and Participants:**

This retrospective, population-based cohort study was conducted between January 1, 2015, and June 30, 2019, in Victoria, Australia using linked EMS, emergency department (ED), hospital, and state death index datasets. Children younger than 18 years who contacted EMS for nontraumatic chest pain were included. Data analyses were completed on July 16, 2025.

**Exposure:**

EMS attendance for nontraumatic chest pain.

**Main Outcomes and Measures:**

The primary end point was a serious outcome within 72 hours, defined as death, cardiac arrest, ED triage category 1 (resuscitation), or intensive care unit (ICU) admission. Multivariable logistic regression identified factors associated with serious outcomes.

**Results:**

Among 4277 pediatric EMS attendances, the median (IQR) age was 14 (11-16) years, and 2506 (58.6%) were female. The overall incidence was 60.0 (95% CI, 58.0-62.0) per 100 000 person-years, with higher rates in females (67.7 [95% CI, 64.9-70.6] cases per 100 000 person-years), adolescents aged 12 to 17 years (128.0 [95% CI, 123.2-133.1] cases per 100 000 person-years), and children from the most disadvantaged areas (78.4 [95% CI, 73.6-83.6] cases per 100 000 person-years). Most cases (3395 [79.4%]) received time-critical EMS dispatch, 3263 (76.3%) were transported to hospital, and 1586 (59.1%) were triaged as urgent (category 3) in the ED. The most common diagnoses were nonspecific chest pain (1131 patients [42.2%]) and respiratory disorders (476 patients [17.7%]); cardiovascular diagnoses were uncommon (191 patients [7.1%]). Within 72 hours, serious outcomes occurred in 44 patients (1.6%). In multivariable models, abnormal initial vital signs including heart rate (odds ratio [OR], 3.50; 95% CI, 1.75-6.97), systolic blood pressure (OR, 6.47; 95% CI, 1.95-21.48), hypoxemia (OR, 5.73; 95% CI, 2.28-14.39), and reduced consciousness (OR, 6.03; 95% CI, 2.40-15.10) were associated with serious outcomes.

**Conclusions and Relevance:**

In this cohort study of children seeking EMS care for nontraumatic chest pain, most cases were benign and rarely of cardiac origin, and abnormal vital signs at EMS assessment were associated with increased risk of serious outcomes. These findings support the need for improved triage systems and risk stratification to guide safe and appropriate care.

## Key Points

**Question:**

What are the incidence, diagnoses, and outcomes of pediatric patients with nontraumatic chest pain attended by emergency medical services (EMS)?

**Findings:**

In this cohort study of 4277 children, incidence for chest pain was 60.0 cases per 100 000 person-years; most cases were benign and not cardiac in origin. Serious outcomes (death, cardiac arrest, resuscitation, or intensive care unit admission) within 72 hours were rare (1.6%), but children with abnormal vital signs at EMS arrival had an increased risk of serious outcomes and hospital admission.

**Meaning:**

These findings suggest improved triage systems and risk stratification are needed to guide safe and efficient care for children with chest pain.

## Teaser

This cohort study analyzes the incidence, diagnoses, and outcomes of children in Australia attended by emergency medical services for nontraumatic chest pain.

## Introduction

Nontraumatic chest pain is a common problem,^1,2^ but less is known about it in children and adolescents seeking emergency medical services (EMS) care. Chest pain can relate to a variety of underlying causes of cardiac or noncardiac origin,^2,3^ causing concern for both patients and families.^1^ While chest pain in adults may signal serious cardiac conditions, the cause of chest pain in children is often benign and noncardiac in nature.^3,4,5,6,7^ Musculoskeletal disorders are a known leading cause of pediatric chest pain; other causes include gastrointestinal and respiratory disorders. Many cases are idiopathic.^4,6,8,9^

Chest pain in general causes fear in patients, and the perceived knowledge of its association with underlying severe life-threatening conditions may lead to excessive EMS use. Despite being a common concern, the literature on pediatric chest pain presentations to EMS is scant. Recent studies have shed light on chest pain presentations in adult populations^10,11^; however, little is known about the epidemiological profile and outcomes of pediatric patients with chest pain requiring prehospital care. Existing pediatric literature has primarily focused on hospital-based studies, which exclude the prehospital journey and patients who are discharged at scene by paramedics.^3,4,10,11^

In this study, we used a large dataset of EMS cases linked with hospital and death index data to describe the characteristics and outcomes of pediatric patients attended by EMS for nontraumatic chest pain in Victoria, Australia. We described the incidence of nontraumatic chest pain and explored the factors associated with patient outcomes.

## Methods

This retrospective cohort study followed the Strengthening the Reporting of Observational Studies in Epidemiology (STROBE) reporting guideline.^12^ Ethics approval was obtained from the Monash University human research ethics committee. The requirement for informed consent was waived due to analysis of deidentified data.

### Study Design and Population

We conducted a retrospective data linkage study examining pediatric (<18 years) patients with nontraumatic chest pain attended by EMS in Victoria, Australia, between January 1, 2015, and June 30, 2019. EMS patient records were linked to hospital emergency department (ED) and admission datasets as well as state death records. The methodology used for this linkage has been described previously.^13^ We excluded trauma-related cases, patients with missing identifiers, and interhospital transfers.

### Setting

Ambulance Victoria provides a 2-tiered EMS to the state of Victoria, which spans approximately 227 500 km^2^ and has a population of 6.6 million. The EMS comprises advanced life support and intensive care paramedics. Triple Zero Victoria manages call-taking, triaging, and dispatching through the national emergency number (000). In Victoria, there are no specific prehospital guidelines for managing pediatric chest pain. Paramedic care focuses on appropriate triage and symptom relief with oral, inhaled, or intranasal analgesics. Intravenous pain relief is reserved for intensive care paramedics and pediatric patients with severe pain. Funding for EMS is primarily covered by government grants, a membership scheme, and private health insurance, with potential out-of-pocket expenses for uninsured residents.

### Data Collection and Sources

Prehospital electronic patient care record and computer-aided dispatch data were linked with data from the Victorian Ambulance Cardiac Arrest Registry, the Victorian Emergency Minimum Dataset (VEMD), the Victorian Admitted Episodes Dataset (VAED), and the Victorian Death Index dataset. The Victorian Ambulance Cardiac Arrest Registry is an ongoing registry managed by Ambulance Victoria and collates information of all out-of-hospital cardiac arrests attended by EMS in the state.^14^ The VEMD contains deidentified demographic, administrative, and clinical data detailing ED presentations at Victorian public hospitals. The VAED contains clinical and administrative data for all admitted episodes of care at Victoria’s hospitals, rehabilitation centers, extended care facilities, and day procedure centers. The Victorian Death Index provides information regarding the date and cause of death for all deaths registered in Victoria.

### Study Definitions and Outcomes

Patient identification was based on prehospital clinical data, utilizing 2 key criteria: (1) paramedic documentation of pain in the chest in their clinical records or (2) a paramedic final primary or secondary assessment indicating ischemic chest pain, acute coronary syndrome, acute myocardial infarction, pleuritic pain, or angina (eMethods in Supplement 1). Patients were stratified into 3 age groups: 0 to 5 years (young children), 6 to 11 years (middle-age children), and 12 to 17 years (adolescents). Age-specific thresholds for abnormal vital signs on paramedic initial assessment were established in accordance with current clinical practice guidelines (eMethods in Supplement 1). The study included consecutive cases that met the inclusion criteria, allowing multiple entries for patients with recurrent acute chest pain presentations. Incidence rate was calculated based on each patient’s first ambulance attendance. Geographical classification of patient residence utilized the Accessibility and Remoteness Index of Australia, which categorizes locations based on service accessibility.^15^ Due to limited events in remote and very remote areas, these categories were consolidated with the outer regional classification. Socioeconomic status was evaluated using the Index of Relative Socioeconomic Disadvantage, a validated measure derived from national Census data. Index of Relative Socioeconomic Disadvantage scores were stratified into quintiles based on residential postcodes, with the first quintile representing the most disadvantaged areas. ED triage category classifies a patient’s clinical acuity using the Australasian Triage Scale.^16^ Primary hospital diagnosis was categorized according to the *International Statistical Classification of Diseases and Related Health Problems, Tenth Revision (ICD-10)* codes version 9.0 coded as VAED diagnosis if a patient was discharged from hospital and VEMD diagnosis if a patient was discharged from ED (eMethods in Supplement 1). Outcomes were assessed at 72 hours, 30 days, and 90 days post-EMS attendance. The primary end point was the occurrence of a serious outcome, defined as death, cardiac arrest, ED triage category 1 (resuscitation), or intensive care unit admission. Secondary end points included all-cause mortality, all-cause EMS recontact defined as any subsequent Triple Zero Victoria (000) call following the index EMS attendance, and hospital admission, defined as an overnight stay, excluding admissions to ED short stay units, and day-only admissions.

### Statistical Analysis

Analyses were completed on July 16, 2025. Incidence rates per 100 000 person-years were calculated using midyear age– and sex-specific population estimates from the Australian Bureau of Statistics, based on each patient’s first ambulance attendance. We estimated 95% CIs for incidence rates assuming a Poisson distribution, and trends were assessed for significance using the Poisson regression with the log of the population at risk as an offset. For region and remoteness and socioeconomic status quintiles, age-standardized rates were calculated using 5-year age brackets. Baseline and prehospital characteristics were analyzed for all EMS-attended cases. Outcome analyses were assessed in 2 subgroups: (1) transported patients with linked hospital records, forming the primary analysis cohort, and (2) nontransported patients. Results were presented stratified by age groups (0-5, 6-11, and 12-17 years) or transport status. Categorical variables were summarized as frequencies and proportions, with group comparisons performed using the Pearson χ^2^ test. Continuous variables were reported as medians with IQRs and compared using the Wilcoxon rank-sum or Kruskal-Wallis test, as appropriate.

Multivariable logistic regression analyses were conducted to identify factors associated with the occurrence of serious outcome at 72 hours and hospital admission. Clinically relevant prehospital covariates (age, sex, socioeconomic status, medical history, and initial vital signs) were included in the models. Missing data were excluded given small number; exclusion rates are shown in table footnotes. Model validity was assessed using likelihood-ratio tests and the Hosmer-Lemeshow goodness-of-fit test. Results are presented as adjusted odd ratios (ORs) and 95% CIs. All analyses used 2-sided tests with a significance level of *P* < .05 using Stata 18 Statistical Software (StataCorp).

## Results

### Patient Characteristics

Between January 1, 2015, and June 30, 2019, there were 289 984 EMS attendances for children (<18 years). After exclusions, 4277 chest pain presentations from 3717 unique patients (1.5% of pediatric EMS attendances) were included (Figure). The median (IQR) age was 14 (11-16) years, and 2506 patients (58.6%) were female. The age distribution was 249 patients (5.9%) aged 0 to 5 years, 988 patients (23.4%) aged 6 to 11 years, and 3040 patients (72.0%) aged 12 to 17 years. Most patients resided in major cities (3223 patients [75.3%]), and 1110 patients (26.0%) lived in the most disadvantaged areas.

**Figure.**
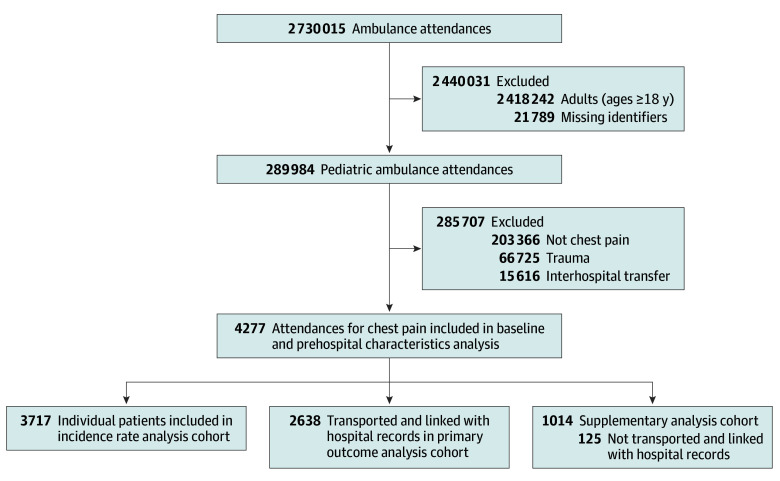
Flowchart of the Study Population

Asthma was the most frequent medical history (1297 patients [30.3%]), particularly among children aged 6 to 11 years (388 patients [39.3%]). Respiratory infections occurred more often in younger children (38 patients aged 0-5 years [15.3%]). EMS dispatch was categorized as potentially time-critical for 3395 cases (79.4%). On arrival, 766 patients (17.9%) had abnormal heart rate, 582 patients (13.6%) had abnormal respiratory rate, and 187 patients (4.4%) had hypoxemia, with higher rates of abnormal vital signs among younger ages.

Prehospital interventions varied by age. Oxygen was administered to 233 patients (5.5%), most frequently among those aged 0 to 5 years (31 patients [12.4%]). Analgesia was provided to 1221 patients (28.6%), predominantly adolescents (979 of 3034 patients [32.2%]). Intravenous access was established in 443 patients (10.4%), and electrocardiogram was performed in 3732 patients (87.3%), increasing by age. A total of 3263 patients (76.3%) were transported to hospital, most commonly in the youngest age group (201 of 249 patients [80.7%]). Time-critical transport occurred in 141 of 3263 patients (4.4%). Detailed patient characteristics are shown in Table 1; missing data proportions by age group are summarized in eTable 1 in Supplement 1.

**Table 1.** Baseline Characteristics of Pediatric Patients With Nontraumatic Chest Pain

Characteristic^a^	Participants by age group, No. (%)				*P* value
	Total (N = 4227)	0-5 y (n = 249)	6-11 y (n = 988)	12-17 y (n = 3040)	
Age, median (IQR), y	14 (11-16)	3 (0-5)	9 (8-10)	16 (14-17)	NA
Sex					
Male	1768 (41.3)	137 (55.0)	477 (48.3)	1154 (38.0)	<.001
Female	2506 (58.6)	112 (45.0)	511 (51.7)	1883 (62.0)	
Accessibility and Remoteness Index of Australia					
Major cities	3223 (75.3)	183 (73.5)	819 (82.9)	2221 (73.1)	<.001
Inner regional	846 (19.8)	51 (20.5)	136 (13.8)	659 (21.7)	
Outer regional or remote	159 (3.7)	13 (5.2)	23 (2.3)	123 (4.1)	
Socioeconomic status quintile (IRSD)					
1 (Most disadvantaged)	1110 (26.0)	64 (25.7)	203 (20.6)	843 (27.7)	.001
2	1018 (23.8)	50 (20.1)	264 (26.7)	704 (23.2)	
3	769 (17.9)	43 (17.3)	179 (18.1)	547 (17.9)	
4	591 (13.8)	32 (12.9)	148 (15.0)	411 (13.5)	
5 (Least disadvantaged)	761 (17.8)	58 (23.3)	190 (19.2)	513 (16.8)	
Medical history					
Asthma	1297 (30.3)	61 (24.5)	388 (39.3)	848 (27.9)	<.001
Respiratory infection	223 (5.2)	38 (15.3)	94 (9.5)	91 (3.0)	<.001
Arrhythmias	221 (5.2)	3 (1.2)	60 (6.1)	158 (5.2)	.008
Hypertension	37 (0.9)	15 (6.0)	4 (0.4)	18 (0.6)	<.001
Diabetes	54 (1.3)	11 (4.4)	7 (0.7)	36 (1.2)	<.001
Coronary disease	33 (0.8)	16 (6.4)	4 (0.4)	13 (0.4)	<.001
Initial prehospital vital signs					
Abnormal heart rate	766 (17.9)	97 (39.0)	150 (15.2)	519 (17.1)	<.001
Abnormal respiratory rate	582 (13.6)	92 (37.0)	143 (14.5)	347 (11.4)	<.001
Abnormal systolic blood pressure	49 (1.1)	<5	9 (0.9)	39 (1.3)	<.001
Abnormal oxygen saturation	187 (4.4)	48 (19.3)	98 (9.9)	41 (1.4)	<.001
Abnormal temperature (≥38 °C)	391 (9.1)	64 (25.7)	125 (12.7)	202 (6.6)	<.001
Reduced consciousness^b^	125 (2.9)	23 (9.2)	14 (1.4)	88 (2.9)	<.001
EMS dispatch priority					
Time critical (lights and sirens response)	3395 (79.4)	195 (78.3)	803 (81.3)	2397 (78.9)	.004
Urgent	747 (17.5)	28 (19.3)	168 (17.0)	531 (17.5)	
Nonurgent	133 (3.1)	6 (2.4)	16 (1.6)	111 (3.7)	
Prehospital treatment					
Oxygen	233 (5.5)	31 (12.4)	84 (8.5)	118 (3.9)	<.001
Analgesia	1221 (28.6)	52 (20.9)	190 (19.2)	979 (32.2)	<.001
Intravenous access^c^	443 (10.4)	26 (10.4)	9 (0.9)	408 (13.4)	<.001
Electrocardiogram performed	3732 (87.3)	173 (69.5)	802 (81.2)	2757 (90.7)	<.001
EMS transport					
Transported	3263 (76.3)	201 (80.7)	720 (72.9)	2342 (77.0)	.007
Not transported	1014 (23.7)	48 (19.3)	268 (27.1)	698 (23.0)	
EMS highest transport code^d^					
Code 1 (lights and sirens)	141 (4.4)	16 (8.0)	36 (5.0)	89 (3.8)	.006
Code 2 (urgent transport without lights and sirens)	2862 (88.6)	178 (89.4)	639 (89.5)	2045 (88.3)	
Code 3 (nonurgent transport)	226 (7.0)	5 (2.5)	39 (5.4)	182 (7.9)	
Time metrics, median (IQR), min					
Call to scene arrival	11.2 (8.4-16.6)	11.5 (8.0-15.7)	11.0 (8.4-16.1)	11.2 (8.4-17.0)	.68
Scene arrival to scene depart	18 (13-28)	17 (12-26)	18 (13-28)	18 (13-28)	.13
Scene depart to hospital arrival	18 (11-27)	19 (12-28)	20 (12-28)	18 (10-27)	.008

Abbreviations: EMS, emergency medical service; IRSD, Index of Relative Socioeconomic Disadvantage; NA, not applicable.

^a^ Missing data for sex (3 participants [0.1%]), Accessibility and Remoteness Index of Australia (49 participants [1.1%]), socioeconomic status (28 participants [0.7%]), abnormal heart rate (16 participants [0.4%]), abnormal respiratory rate (16 participants [0.4%]), abnormal systolic blood pressure (223 participants [5.2%]), abnormal oxygen saturation (118 participants [2.8%]), abnormal temperature (309 participants [7.2%]), and reduced consciousness (10 participants [0.2%]).

^b^ According to the Glasgow Coma Scale (<13 for those aged 16 years or older and <15 for those aged younger than 16 years).

^c^ Intravenous access in children younger than 12 years is only attempted in cardiac arrest, as per clinical practice guidelines.

^d^ As a proportion of transported patients.

### Transported vs Nontransported Patients

Of the 4277 cases, 3263 (76.3%) were transported and 1014 (23.7%) were not (eTable 2 in Supplement 1). Transported patients were slightly older (median [IQR] age, 15 [11-16] years vs 14 [10-16] years) and more frequently had asthma (1017 patients [31.2%] vs 280 patients [27.6%]), arrhythmias (203 patients [6.2%] vs 18 patients [1.8%]), abnormal heart rate (705 patients [21.6%] vs 61 patients [6.0%]), and hypoxemia (171 patients [5.2%] vs 16 patients [1.6%]). Prehospital interventions including oxygen (230 patients [7.1%] vs 3 patients [0.3%]), analgesia (1164 patients [35.7%] vs 57 patients [5.6%]), intravenous access (443 patients [13.6%] vs 0 patients), and electrocardiogram (2916 patients [89.4%] vs 816 patients [80.5%]) were more common among transported patients.

### Linked and Unlinked Transported Patients

Among 3263 transported patients, 2683 (82.2%) could be matched to hospital records, while 580 (17.8%) could not be matched (eTable 3 in Supplement 1). Demographics, prehospital management, and transport times were similar between matched and unmatched patients. Unmatched patients more often resided in outer regional or remote areas (45 patients [7.8%] vs 86 patients [3.2%]) and the most disadvantaged areas (167 patients [28.8%] vs 680 patients [25.3%]) and had higher rates of abnormal heart rate (157 patients [27.1%] vs 548 patients [20.4%]) and respiratory rate (122 patients [21.0%] vs 353 patients [13.2%]). Other baseline characteristics were similar across both groups.

### Incidence

The incidence of EMS attendance for pediatric chest pain was 60.0 (95% CI, 58.0-62.0) cases per 100 000 person-years (Table 2 and the eFigure in Supplement 1). Incidence was higher in females (67.7 [95% CI, 64.9-70.6] cases per 100 000 person-years), adolescents (128.0 [95% CI, 123.2-133.1] cases per 100 000 person-years), and major cities (60.2 [95% CI, 58.0-62.5] cases per 100 000 person-years). Incidence was greatest in the most disadvantaged areas (78.4 [95% CI, 73.6-83.6] cases per 100 000 person-years) and lowest in the least disadvantaged areas (48.3 [95% CI, 44.7-52.1] cases per 100 000 person-years). Annual incidence fluctuated between 61.1 (95% CI, 57.0-65.4) cases per 100 000 person-years in 2015, 54.2 (95% CI, 50.5-58.3) cases per 100 000 person-years in 2017, and 65.0 (95% CI, 59.4-71.3) cases per 100 000 person-years in 2019 (eTable 4 in Supplement 1).

**Table 2.** Incidence of Ambulance Attendance for Pediatric Chest Pain Over the Study Period (2015-2019)

Characteristics	Ambulance attendance, incidence per 100 000 person-years (95% CI)
Overall	60.0 (58.0-62.0)
Sex	
Male	51.7 (49.2-54.3)
Female	67.7 (64.9-70.6)
Age group, y	
0-5	11.4 (10.1-13.0)
6-11	44.4 (41.6-47.4)
12-17	128.0 (123.2-133.1)
Accessibility and Remoteness Index of Australia	
Major cities	60.2 (58.0-62.5)
Inner regional	53.1 (49.4-57.2)
Outer regional or remote	58.9 (50.0-69.3)
Socioeconomic status quintile (Index of Relative Socioeconomic Disadvantage)	
1 (Most disadvantaged)	78.4 (73.6-83.6)
2	68.7 (64.4-73.3)
3	62.5 (58.0-67.4)
4	50.0 (45.8-54.6)
5 (Least disadvantaged)	48.3 (44.7-52.1)

### Primary Analysis

Table 3 shows the results of the primary analysis. Of 2683 transported patients with matched hospital records, 2667 (99.4%) underwent ED triage and 1586 (59.1%) were classified as urgent (category 3). The median (IQR) ED length of stay was 3.1 (2.0-4.5) hours. Most were discharged home (1655 patients [61.7%]), with 362 (13.5%) admitted to a ward and 471 (17.6%) to short stay units. Hospital admission occurred in 194 patients (7.2%; median [IQR] length of stay, 3 [2-5] days). Intensive care unit admissions were rare (27 patients [1.0%]). At 72 hours, 44 patients (1.6%) experienced a serious outcome, and 101 patients (3.8%) had EMS recontact. Recontact increased to 386 patients (14.4%) at 30 days and 628 patients (23.4%) at 90 days, with higher rates among adolescents. Mortality was rare at all time points. Final diagnoses included nonspecific chest pain in 1131 patients (42.2%), most common in adolescents (869 of 1953 patients [44.5%]); respiratory disorders in 476 patients (17.7%), particularly in the youngest group (44 of 115 patients [38.3%]); and cardiovascular disease (including arrhythmias) in 191 patients (7.1%) (eFigure in Supplement 1).

**Table 3.** Patient Outcomes

Outcome	Participants by age group, No. (%)				*P* value
	Total (n = 2683)^a^	0-5 y (n = 115)	6-11 y (n = 615)	12-17 y (n = 1953)	
ED presentation	2667 (99.4)	114 (99.1)	613 (99.7)	1940 (99.3)	.58
ED triage category					
Resuscitation	16 (0.6)	<5	<5	11 (0.6)	.52
Emergency	450 (16.8)	23 (20.0)	97 (15.8)	330 (16.9)	
Urgent	1586 (59.1)	64 (55.7)	361 (58.7)	1161 (59.5)	
Semiurgent or nonurgent	615 (22.9)	25 (21.7)	152 (24.7)	438 (22.4)	
ED length of stay, median (IQR), h	3.1 (2.1-4.5)	3.2 (2.1-4.6)	2.9 (1.9-4.1)	3.2 (2.1-4.5)	<.001
ED discharge destination					
Home	1655 (61.7)	60 (52.2)	398 (64.7)	1197 (61.3)	.001
Short stay unit	471 (17.6)	21 (18.3)	88 (14.3)	362 (18.5)	
To ward	362 (13.5)	28 (24.4)	92 (15.0)	242 (12.4)	
Other	179 (6.7)	5 (4.4)	35 (5.7)	139 (7.1)	
In-hospital outcomes					
Admitted	194 (7.2)	5 (4.3)	38 (6.1)	151 (7.7)	.20
Hospital length of stay, median (IQR), d	3 (2-5)	3 (2-3)	2 (2-3)	3 (2-5)	.04
ICU admission	27 (1.0)	<5	7 (1.1)	18 (0.9)	.64
ICU length of stay, median (IQR), h	31 (17-72)	79.5 (17-142)	21 (16-67)	37.5 (17-80)	.60
Final diagnosis category					
Cardiovascular	191 (7.1)	3 (2.6)	47 (7.6)	141 (7.2)	<.001
Respiratory disease	476 (17.7)	44 (38.3)	177 (28.8)	255 (13.1)	
Gastroenterology	70 (2.6)	0	14 (2.3)	56 (2.9)	
Mental health	162 (6.0)	0	7 (1.1)	155 (7.9)	
Musculoskeletal	126 (4.7)	2 (1.7)	22 (3.6)	102 (5.2)	
Nonspecific chest pain	1131 (42.2)	36 (31.3)	226 (36.8)	869 (44.5)	
Other specialties	370 (13.8)	22 (19.1)	88 (14.3)	260 (13.3)	
72-h Outcomes					
Serious outcome	44 (1.6)	<5	10 (1.6)	32 (1.6)	.91
All-cause mortality	<5	0	0	<5	.83
EMS recontact	101 (3.8)	<5	18 (2.9)	82 (4.2)	.08
30-d Outcomes					
All-cause mortality	<5	0	0	<5	.68
EMS recontact	386 (14.4)	7 (6.1)	66 (10.7)	313 (16.0)	<.001
90-d Outcomes					
All-cause mortality	<5	0	0	<5	.37
EMS recontact	628 (23.4)	13 (11.3)	108 (17.6)	507 (26.0)	<.001

Abbreviations: EMS, emergency medical service; ED, emergency department; ICU, intensive care unit.

^a^ Missing data (36 participants [1.3%]).

In multivariable regression (Table 4), abnormal vital signs were associated with increased risk of serious outcome at 72 hours (abnormal heart rate: OR, 3.50; 95% CI, 1.75-6.97; abnormal systolic blood pressure: OR, 6.47; 95% CI, 1.95-21.48; hypoxemia: OR, 5.73; 95% CI, 2.28-14.39; reduced consciousness: OR, 6.03; 95% CI, 2.40-15.10). Factors associated with increased risk of hospital admission included older age (OR, 1.11; 95% CI, 1.05-1.17), male sex (OR, 1.39; 95% CI, 1.02-1.90), higher socioeconomic status (OR, 1.18; 95% CI, 1.06-1.31), abnormal heart rate (OR, 1.68; 95% CI, 1.17-2.40), hypoxemia (OR, 5.82; 95% CI, 3.36-10.06), and reduced consciousness (OR, 2.38; 95% CI, 1.25-4.52).

**Table 4.** Multivariable Analyses of Factors Associated With Serious Outcomes and Hospitalization at 72 Hours^a^

Characteristic^b^	Serious outcome, OR (95% CI)	*P* value	Hospital admission, OR (95% CI)	*P* value
Age	1.08 (0.97-1.20)	.14	1.11 (1.05-1.17)	<.001
Male sex	1.87 (0.97-3.60)	.06	1.39 (1.02-1.90)	.03
Prior arrhythmias	2.33 (0.87-6.27)	.09	0.51 (0.23-1.13)	.09
Socioeconomic status^c^	1.13 (0.90-1.42)	.30	1.18 (1.06-1.31)	.003
Vital signs				
Abnormal initial heart rate	3.50 (1.75-6.97)	<.001	1.68 (1.17-2.40)	.004
Abnormal initial respiratory rate	2.10 (0.99-4.47)	.05	0.99 (0.64-1.55)	.98
Abnormal initial systolic blood pressure	6.47 (1.95-21.48)	.002	0.73 (0.17-3.18)	.67
Abnormal initial oxygen saturation	5.73 (2.28-14.39)	<.001	5.82 (3.36-10.06)	<.001
Reduced consciousness^d^	6.03 (2.40-15.10)	.001	2.38 (1.25-4.52)	.008

^a^ A total of 2516 observations were included in each model.

^b^ Missing data for socioeconomic status (11 participants [0.4%]), abnormal heart rate (5 participants [0.2%]), abnormal respiratory rate (6 participants [0.2%]), abnormal systolic blood pressure (112 participants [4.2%]), abnormal oxygen saturation (51 participants [1.0%]), and reduced consciousness (1 participant [<0.0%]).

^c^ Socioeconomic status used the Index of Relative Socioeconomic Disadvantage decile.

^d^ According to the Glasgow Coma Scale (<13 for those aged 16 years or older; <15 for those aged younger than 16 years).

### Supplementary Analysis: Nontransported Patients

Among 1014 nontransported patients, 120 (11.8%) subsequently presented to an ED (eTable 5 in Supplement 1). Of these, 62 (51.7%) were triaged as urgent, and 82 (68.3%) were discharged home, often with nonspecific chest pain (45 patients [40.5%]). Hospital admission occurred in 5 patients (4.4%); no intensive care unit admissions were recorded. EMS recontact occurred in fewer than 5 patients at 72 hours and increased to 16 patients (13.3%) at 30 days and 25 patients (20.8%) at 90 days. Serious outcomes and mortality remained rare.

## Discussion

In this population-based cohort study of EMS-attended, pediatric, nontraumatic chest pain, the incidence was 60.0 cases per 100 000 person-years, with greater rates among females, older children, and those from disadvantaged areas. Most patients received no specific diagnosis, and when identified, respiratory disorders were the most frequent cause of chest pain; cardiovascular causes were uncommon. Despite frequent urgent EMS dispatch and high hospital transport rates, hospital admission, all-cause EMS recontact, and serious outcomes remained uncommon. However, children with abnormal vital signs at EMS arrival had increased odds of serious outcomes at 72 hours and of hospital admission.

To our knowledge, this is the first population-wide study to include all EMS-attended, pediatric, nontraumatic chest pain, capturing both patients transported and those discharged at the scene. Our findings align with and extend prior hospital-based research, which report that cardiac causes account for only a minority of pediatric chest pain cases, typically no more than 10%, and the vast majority are generally benign.^1,2,4,6,7,8,9,17^ A recent systematic review assessing the presentation, risk factors, and outcomes of pediatric chest pain in the ED found only 2.5% of presentations were cardiac, with most being musculoskeletal, idiopathic, or gastrointestinal in origin.^4^

The observed prevalence of respiratory diagnoses and the unspecified diagnoses in our cohort highlights the diagnostic challenges facing in this population, stemming from the nonspecific presentation of chest pain in children, age-related communication barriers or limitations in current diagnostic tools.^18,19^ These results suggests the need for clear, standardized clinical pathways and risk stratification tools specific to pediatric chest pain to improve assessment accuracy and avoid unnecessary escalation of care.^5,20,21^

The hospital admission rate observed in our cohort was higher than rates reported in ED-based studies,^4,17,22^ likely reflecting the greater acuity or perceived severity among patients attended by EMS.^23^ This finding suggests that EMS patients represent a higher-risk subset, even if the ultimate risk for serious outcome is low. Our finding affirms the critical role of EMS assessment in identifying patients who require further hospital evaluation vs those who could safely be treated through alternative care pathways.

The higher incidence of EMS-attended chest pain in socioeconomically disadvantaged areas is consistent with broader evidence of the influence of social determinants on emergency health care utilization.^24,25^ Although financial barriers often reduce access to health services, the increased EMS use in these groups may reflect unmet health needs, higher disease burden, or reduced access to primary care pathways. This socioeconomic gradient highlights opportunities for targeted preventive strategies and interventions to improve access and health equity.

Optimizing care for pediatric chest pain in prehospital and emergency settings requires a balance between thorough evaluation and the avoidance of unnecessary hospital utilization. In our cohort, most EMS responses were classified as time-critical; however, the actual occurrence of serious outcomes, prolonged ED stays, and hospital admissions were low. This finding suggests that many children could potentially be treated safely outside of hospital settings, provided that robust triage system and outpatient referral pathways are in place. Nevertheless, EMS and ED care remain crucial for identifying and escalating care for the truly high-risk minority and for excluding life-threatening conditions, particularly among children presenting with deranged vital signs. Future research is needed to validate pediatric-specific risk stratification models and to develop safe alternative care pathways, such as outpatient clinics or telemedicine consultations for low-risk children.

### Limitations

This study has several limitations. Our cohort was limited to pediatric patients who contacted EMS for nontraumatic chest pain and excluded cases presenting directly to ED or other health services. Consequently, our incidence rates reflect EMS attendances rather than the total population burden of pediatric chest pain, a cohort which may have different acuity and underlying diagnoses. While financial barriers such as out-of-pocket costs for uninsured patients may influence EMS utilization and contribute to the observed variation in incidence across socioeconomic groups, our findings showed higher EMS use among disadvantaged groups, likely reflecting greater illness burden or reduced access to alternative care pathways. Due to the linkage technique used, 17.8% of transported patients were unable to be successfully matched to the VAED and VEMD registries and were therefore not included in our outcome analyses, which could represent a potential source of selection bias because these unmatched patients may differ from those with linked records. Additionally, some clinical variables in EMS records rely heavily on self-reporting or caregiver information, potentially introducing bias, especially in pediatric cases where obtaining accurate medical history can be challenging. The low number of serious outcomes and hospital admission in our cohort necessitated limiting the number of covariates in regression analyses to avoid overfitting. As a result, some estimates are associated with wide confidence intervals, and these should be interpreted with caution. Additionally, while the Index of Relative Socioeconomic Disadvantage provides valuable insights into socioeconomic factors, it focuses on indicators of disadvantage and describes area-level, not individual, socioeconomic status. The interpretation of this variable should be considered with this limitation in mind.

## Conclusions

In this cohort study, pediatric nontraumatic chest pain attended by EMS was infrequent and rarely due to cardiac causes. Most cases were benign despite frequent urgent EMS responses and hospital transport. Abnormal vital signs at EMS assessment were associated with increased risk of serious outcomes and hospital admission. These findings support the need for targeted prehospital assessment and pediatric-specific risk stratification guidelines to optimize emergency care and reduce unnecessary hospital utilization for children presenting with chest pain.

## Notes

Supplement 1.
eMethods.
**eTable 1.** Proportion of Missing Data for Prehospital Variables **eTable 2.** Baseline Characteristics of Pediatric Patients with Non-Traumatic Chest Pain Stratified by Transport **eTable 3.** Baseline Characteristics of Transported Patients, Stratified by Linkage to Hospital Records **eFigure.** Age- and Sex-Specific Incidence Rates of Chest Pain Attendances per 100,000 Person-Years (A) and Final Primary Diagnosis Among Ambulance Attendances for Pediatric Chest Pain (B) **eTable 4.** Annual Incidence of Ambulance Attendances for Chest Pain Per 100,000 Population Across the Study Period **eTable 5.** Outcomes of Non-Transported Patients, Stratified by Age

Supplement 2.
Data Sharing Statement
