# Supplementary material for: Incidence, Diagnoses, and Outcomes of Pediatric Nontraumatic Chest Pain Attended by Ambulance
Source: JAMA Netw Open. 2025 Sep 26;8(9):e2533962. doi: 10.1001/jamanetworkopen.2025.33962 (PMC12475941; doi:10.1001/jamanetworkopen.2025.33962)
Supplement: Supplement 1. — eMethods. eTable 1. Proportion of Missing Data for Prehospital Variables eTable 2. Baseline Characteristics of Pediatric Patients with Non-Traumatic Chest Pain Stratified by Transport eTable 3. Baseline Characteristics of Transported Patients, Stratified by Linkage to Hospital Records eFigure. Age- and Sex-Specific Incidence Rates of Chest Pain Attendances per 100,000 Person-Years (A) and Final Primary Diagnosis Among Ambulance Attendances for Pediatric Chest Pain (B) eTable 4. Annual Incidence of Ambulance Attendances for Chest Pain Per 100,000 Population Across the Study Period eTable 5. Outcomes of Non-Transported Patients, Stratified by Age [file jamanetwopen-e2533962-s001.pdf]

Supplemental Online Content

Okyere D, Nehme E, Mahony E, et al. Incidence, diagnoses, and outcomes of pediatric nontraumatic chest pain attended by ambulance. *JAMA Netw. Open*. 2025;8(9):e2533962. doi:10.1001/jamanetworkopen.2025.33962

eMethods.

eTable 1. Proportion of Missing Data for Prehospital Variables

eTable 2. Baseline Characteristics of Pediatric Patients with Non-Traumatic Chest Pain Stratified by Transport

eTable 3. Baseline Characteristics of Transported Patients, Stratified by Linkage to Hospital Records

eFigure. Age- and Sex-Specific Incidence Rates of Chest Pain Attendances per 100,000 Person-Years (A) and Final Primary Diagnosis Among Ambulance Attendances for Pediatric Chest Pain (B)

eTable 4. Annual Incidence of Ambulance Attendances for Chest Pain Per 100,000 Population Across the Study Period

eTable 5. Outcomes of Non-Transported Patients, Stratified by Age

This supplemental material has been provided by the authors to give readers additional information about their work.

© 2025 Okyere D et al. *JAMA Network Open*.

eMethods.

Identification of Chest Pain Cases:

The below describes the systematic search strategy and regular expression logic used to identify cases of chest pain from electronic prehospital Patient care records.

1. **Paramedic Final Primary Assessment:** "Pain :Location :Ischaemic Chest", "Acute Coronary Syndrome", "Acute Myocardial Infarction", "Pain :Location :Pleuritic Chest", "Angina", "Ami ", "Stemi", "Nstemi", "Chest Pain", "Ccp", "Nsteacs"

2. **Final secondary Assessment:** "Pain :Location :Ischaemic Chest", "Acute Coronary Syndrome", "Acute Myocardial Infarction", "Pain :Location :Pleuritic Chest", "Angina", "Ami ", "Stemi", "Nstemi", "Chest Pain", "Ccp", "Nsteacs"

3. **Paramedic Secondary Survey Summary**: "Pain :" AND ("Chest", "Cp", "Ccp", "Pic ", "Sternal", "sternal")

   Cases were excluded if any of a list of negating expressions appeared, including: "Nil Chest", "No Chest", "Denies Chest", "Denied Chest", "Nil Cp", "No Cp", "Denies Cp", "Denied Cp", "Nil Ccp", "No Ccp", "Denies Ccp", "Denied Ccp", "Nil Pic ", "No Pic ", "Denies Pic ", "Denied Pic ", "Pic Line", "No Sternal", "Nil Sternal", "Denies Sternal", "Denied Sternal", "No Retrosternal", "Nil Retrosternal", "Denies Retrosternal", "Denied Retrosternal", "No Retro-Sternal", "Nil Retro-Sternal", "Denies Retro-Sternal", "Denied Retro-Sternal", "Denies Any Chest Pain", "Denied Any Chest Pain", "Denies Any Cp", "Denied Any Cp"

Primary hospital diagnosis

Final diagnoses for patients were categorized in accordance with International Classification of Diseases [ICD] 10 V9.0 codes as follows

1. Cardiovascular: I00-I99
2. Respiratory disease: J00-J998
3. Gastroenterology: K000-K938
4. Other specialties: A000-E899, G000-H959, L00-Q999, S001-Z999
5. Mental health: F00-F99
6. Non-specific chest pain: R000-R99

© 2025 Okyere D et al. *JAMA Network Open*.

Age-specific vital sign derangement criteria

Age	Initial Pulse Rate (beats/min)	Initial Respiratory Rate (breath/min)	Initial BP Systolic (mmHg)	Glasgow Coma Scale	Oxygen Saturation (%)
Newborn (<24 hours)	<110 or >170	<25 or >60	<60	<15	<96
Small infant (<3 months)	<110 or >170	<25 or >60	<60	<15	<96
Large infant (3-12 months)	<105 or >165	<25 or >55	<65	<15	<96
Small child (1-4 years)	<85 or >150	<20 or >40	<70	<15	<96
Medium child (5-11 years)	<70 or >135	<16 or >34	<80	<15	<96
12-17 years	>120	>30	<90	<13 (<15 if age <16)	<90

© 2025 Okyere D et al. *JAMA Network Open*.

eTable 1.Proportion of Missing Data for Prehospital Variables

Missing Data, n (%)	**Total (N= 4227)**	**0-5 years (N=249)**	**6-11 years (N= 988)**	**12-17 years (N= 3040)**
Age (years)	0	**0**	**0**	**0**
Sex	3 (0.1)	0	0	3 (0.1)
Accessibility and Remoteness Index of Australia (ARIA)	49 (1.2)	2 (0.8)	10 (1.0)	37 (1.2)
Socioeconomic status (IRSD)	28 (0.6)	2 (0.8)	4 (0.4)	22 (0.7)
Medical history	3 (0.1)	0	0	3 (0.1)
Initial prehospital vital signs				
Abnormal Heart Rate, beats/min	16 (0.4)	5 (2.0)	1 (0.1)	10 (0.3)
Abnormal Respiratory Rate, breaths/min	16 (0.4)	3 (1.2)	2 (0.2)	11 (0.4)
Abnormal Systolic Blood Pressure, mm Hg	223 (5.2)	83 (33.3)	109 (11.0)	31 (1.0)
Abnormal Oxygen Saturation	118 (2.7)	23 (9.2)	27 (2.7)	68 (2.2)
Abnormal Temperature (>=38), °C	309 (7.2)	25 (10.0)	57 (5.8)	227 (7.5)
Reduced consciousness	10 (0.2)	2 (0.8)	0	8 (0.3)
EMS dispatch priority	2 (0.1)	0	1 (0.1)	1 (0.03)
Prehospital treatment				
Oxygen	0	0	0	0
Analgesia	0	0	0	0
Intravenous access[a]	0	0	0	0
ECG performed	0	0	0	0
EMS Transport	0	0	0	0
Time metrics				
Call to scene arrival	49 (1.1)	1 (0.4)	9 (0.9)	39 (1.3)
Scene arrival to scene depart	5 (0.1)	0	1 (0.1)	4 (0.1)
Scene depart to hospital arrival	19 (0.6)	2 (1.0)	5 (0.7)	12 (0.5)

Abbreviations: EMS, Emergency Medical Response; IQR, Interquartile range; IRSD, Index of Relative Socioeconomic Disadvantage.
a=Intravenous access in children <12 years is only attempted in cardiac arrest, as per clinical practice guidelines

© 2025 Okyere D et al. *JAMA Network Open*.

eTable 2. Baseline Characteristics of Pediatric Patients with Non-Traumatic Chest Pain Stratified by Transport				
**Variable Name**	**Total (N= 4227)**	**Transport (N=3263)**	**Non-transport (N=1014)**	**P-Value**
Age, median (IQR), years	14 (11, 16)	15 (11, 16)	14 (10, 16)	0.01
Sex, n (%)				
Male	1768 (41.3)	1366 (41.9)	402 (39.6)	0.21
Female	2506 (58.6)	1895 (58.1)	611 (60.3)	
Accessibility and Remoteness Index of Australia (ARIA), n (%)				0.001
Major Cities	3223 (75.4)	2427 (74.4)	796 (78.5)	
Inner Regional	846 (19.8)	675 (20.7)	171 (16.9)	
Outer Regional / Remote	159 (3.7)	131 (4.0)	28 (2.8)	
Socio-economic status				0.253
Quintile 1 (most disadvantaged)	1110 (26.0)	847 (26.0)	263 (25.9)	
Quintile 2	1018 (23.8)	762 (23.4)	256 (25.3)	
Quintile 3	769 (18.0)	600 (18.4)	169 (16.7)	
Quintile 4	591 (13.8)	439 (13.5)	152 (15.0)	
Quintile 5 (least disadvantaged)	761 (17.8)	596 (18.3)	165 (16.3)	
Medical History, n (%)				
Asthma	1297 (30.3)	1017 (31.2)	280 (27.6)	0.03
Respiratory Infection	223 (5.2)	163 (5.0)	60 (5.9)	0.24
Arrhythmias	221 (5.2)	203 (6.2)	18 (1.8)	<0.001
Hypertension	37 (1.1)	36 (1.3)	1 (0.1)	0.006
Diabetes	54 (1.6)	49 (1.8)	5 (0.7)	0.03
Coronary disease	33 (0.9)	29 (1.1)	4 (0.5)	0.2
Initial prehospital vital signs, n (%)				
Abnormal Heart Rate, beats/min	766 (17.9)	705 (21.6)	61 (6.0)	<0.001
Abnormal Respiratory Rate, breaths/min	582 (13.6)	475 (14.6)	107 (10.6)	<0.001
Abnormal Systolic Blood Pressure, mm Hg	49 (1.2)	47 (1.4)	2 (0.2)	0.001
Abnormal Oxygen Saturation, %	187 (4.4)	171 (5.2)	16 (1.6)	<0.001
Abnormal Temperature (>=38°C)	391 (9.1)	324 (9.9)	67 (6.6)	0.002
Reduced consciousness	125 (2.9)	119 (3.6)	6 (0.6)	<0.001
EMS dispatch priority , n (%)				0.103
Time critical (lights and sirens response)	3395 (79.4)	2616 (80.2)	779 (76.8)	
Urgent	747 (17.5)	546 (16.7)	201 (19.8)	
Non-Urgent	133 (3.1)	99 (3.0)	34 (3.4)	
Prehospital treatment, n (%)				
Oxygen	233 (5.4)	230 (7.1)	3 (0.3)	<0.001
Analgesic	1221 (28.6)	1164 (35.7)	57 (5.6)	<0.001
Intravenous access[a]	443 (10.4)	443 (13.6)	0 (0.0)	<0.001
ECG performed	3732 (87.2)	2916 (89.4)	816 (80.5)	<0.001

© 2025 Okyere D et al. *JAMA Network Open*.

Time metrics (mins), median (IQR)				
Call to scene arrival	11.2 (8.4, 16.6)	11.1 (8.3, 16.2)	11.5 (8.6, 17.7)	0.003
Scene arrival to scene depart	18 (13, 28)	16 (12, 21)	35 (26, 47)	<0.001

Abbreviations: EMS, Emergency Medical Service, IQR, Interquartile range

a=Intravenous access in children <12 years is only attempted in cardiac arrest, as per clinical practice guidelines

Missing data for Sex: 3 (0.1%), ARIA: 49 (1.1%), Socio-economic status: 28 (0.7%), Abnormal Heart rate: 16 (0.4%), Abnormal Respiratory rate: 16 (0.4%), Abnormal Systolic Blood Pressure: 223 (5.2%), Abnormal Oxygen Saturation: 118 (2.8%) , Abnormal Temperature: 309 (7.2%), Reduced consciousness according to Glasgow Coma Scale (<13 (<15 if age <16)): 10 (0.23%)

© 2025 Okyere D et al. *JAMA Network Open*.

eTable 3.Baseline Characteristics of transported patients, stratified by linkage to hospital records

Variable Name	Total (N= 3,263)	Linked cohort (N=2683)	Unlinked cohort (N=580)	P-Value
Age, median (IQR), years	15 (11, 16)	15 (11, 16)	14 (9, 16)	0.08
Sex, n (%)				0.08
Male	1366 (41.9)	1117 (41.6)	249 (42.9)	
Female	1895 (58.1)	1566 (58.4)	329 (56.7)	
Accessibility and Remoteness Index of Australia (ARIA), n (%)				<0.001
Major Cities	2427 (74.4)	2051 (76.4)	376 (64.8)	
Inner Regional	675 (20.7)	530 (19.8	145 (25.0)	
Outer Regional / Remote	131 (4.0)	86 (3.2)	45 (7.8)	
Socio-economic status (IRSD), n (%)				
Quintile 1 (most disadvantaged)	847 (26.0)	680 (25.3)	167 (28.8)	0.027
Quintile 2	762 (23.4)	632 (23.6)	130 (22.4)	
Quintile 3	600 (18.4)	508 (18.9)	92 (15.9)	
Quintile 4	439 (13.4)	363 (13.5)	76 (13.1)	
Quintile 5 (least disadvantaged)	596 (18.3)	489 (18.2)	107 (18.5)	
Medical History, n (%)				
Asthma	1,017 (31.2)	867 (32.3)	150 (25.9)	0.002
Respiratory Infection	163 (5.0)	136 (5.1)	27 (4.7)	0.678
Arrhythmias	203 (6.2)	178 (6.6)	25 (4.3)	0.036
Hypertension	36 (1.3)	16 (0.7)	20 (4.2)	<0.001
Diabetes	49 (1.8)	36 (1.6)	13 (2.8)	0.08
Coronary disease	29 (1.1)	13 (0.6)	16 (3.4)	<0.001
Initial prehospital vital signs, n (%)				
Abnormal Heart Rate, beats/min	705 (21.6)	548 (20.4)	157 (27.1)	0.001
Abnormal Respiratory Rate, breaths/min	475 (14.6)	353 (13.2)	122 (21.0)	<0.001
Abnormal Systolic Blood Pressure, mm Hg	47 (1.4)	33 (1.2)	14 (2.4)	0.08
Abnormal Oxygen Saturation, %	171 (5.2)	138 (5.1)	33 (5.7)	0.05
Abnormal Temperature (>=38°C)	324 (9.9)	272 (10.1)	52 (9.0)	0.23
Reduced consciousness	119 (3.7)	91 (3.4)	28 (4.8)	0.22
EMS dispatch priority , n (%)				
Time critical (lights and sirens response)	2,616 (80.2)	2,137 (79.7)	479 (82.6)	0.239
Urgent	546 (16.7)	461 (17.2)	85 (14.7)	
Non-Urgent	99 (3.0)	84 (3.1)	15 (2.6)	
Prehospital treatment, n (%)				
Oxygen	230 (7.1)	192 (7.2)	38 (6.6)	0.6
Analgesic	1,164 (35.7)	979 (36.5)	185 (31.9)	0.04
Intravenous access	443 (13.6)	364 (13.6)	79 (13.6)	0.97
ECG performed	2,916 (89.4)	2,395 (89.3)	521 (89.8)	0.69
Time metrics (mins), median (IQR)				

© 2025 Okyere D et al. *JAMA Network Open*.

Call to scene arrival	11 (8.3, 16.2)	10 (8.3, 16.1)	11.1 (8.3, 17.0)	0.48
Scene arrival to scene depart	16 (12, 21)	16 (12, 21)	16 (12, 22)	0.07
Scene depart to hospital arrival	20 (14, 28)	20 (14, 29)	18 (12, 28)	0.003

Abbreviations: EMS, Emergency Medical Service, IQR, Interquartile range

a=Intravenous access in children <12 years is only attempted in cardiac arrest, as per clinical practice guidelines

Missing data for Sex: 2 (0.06%), ARIA: 30 (0.92%), Socio-economic status: 19 (0.58%), Abnormal Heart rate: 8 (0.25%), Abnormal Respiratory rate: 7 (0.21%), Abnormal Systolic Blood Pressure: 139 (4.26%), Abnormal Oxygen Saturation: 71 (2.8%) , Abnormal Temperature: 222 (6.80%), Reduced consciousness according to Glasgow Coma Scale (<13 (<15 if age <16)): 1 (0.03%)

© 2025 Okyere D et al. *JAMA Network Open*.

eFigure. Age- and Sex-Specific Incidence Rates of Chest Pain Attendances per 100,000 Person-Years (A) and Final Primary Diagnosis Among Ambulance Attendances for Pediatric Chest Pain (B)

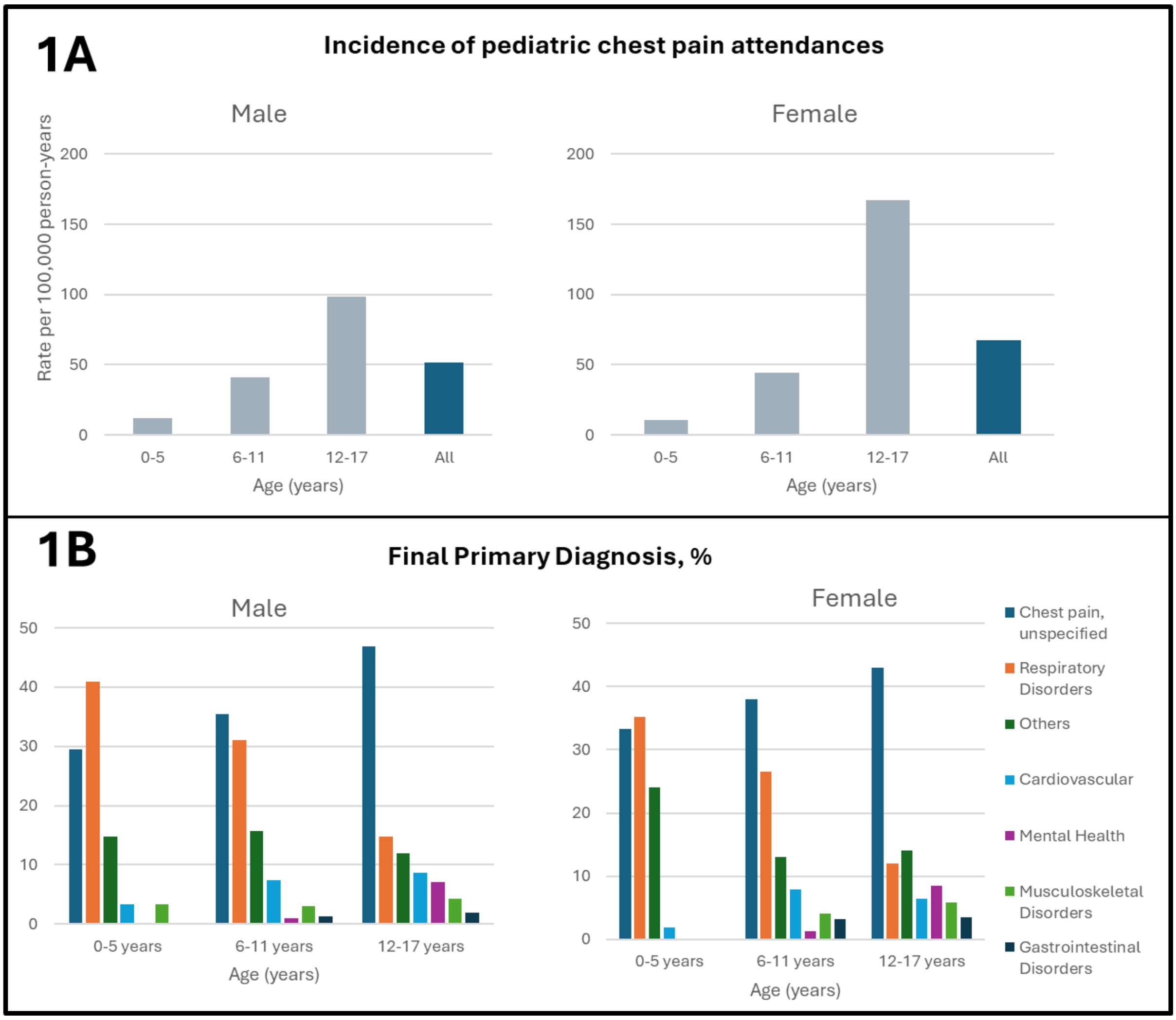

© 2025 Okyere D et al. *JAMA Network Open*.

**eTable 4. Annual incidence of ambulance attendances for chest pain per 100,000 population across the study period**						
**Incidence / 100,000 population (95% CI)**						
	**2015**	**2016**	**2017**	**2018**	**2019[a]**	**$P_{for\ trend}$**
**Overall**						
All	61.1 (57.0-65.4)	59.9 (55.9-64.1)	54.2 (50.5-58.3)	61.9 (57.9-66.2)	65.0 (59.4-71.3)	0.38
**Sex**						
Male	47.2 (42.2-52.8)	48.7 (43.7-54.3)	48.5 (43.5-54.1)	59.4 (53.9-65.4)	56.9 (49.4-65.4)	<0.001
Female	73.0 (66.9-79.7)	70.6 (64.6-77.1)	61.6 (56.1-67.7)	63.0 (57.4-69.0)	73.3 (65.0-82.7)	<0.001
**Age (years)**						
0-5	12.0 (9.3 - 15.6)	10.9 (8.4-14.3)	10.3 (7.8-13.6)	12.4 (9.6-15.9)	11.2 (7.7-16.3)	<0.001
6-7	49.2 (43.0-56.2)	42.0 (36.5-48.6)	40.0 (35.0-46.8)	45.0 (39.7-52.0)	46.0 (37.8-55.3)	<0.001
12-17	125.4 (115.2-136.4)	132.0 (121.7-143.2)	119.0 (109.3-129.5)	129.0 (119.0-139.9)	140.9 (126.5-157.0)	<0.001

Poisson regression was used for $P_{for\ trend.}$
[a] includes data up to June 2019.
95% confidence interval (95%CI)

© 2025 Okyere D et al. *JAMA Network Open*.

eTable 5. Outcomes of non-transported patients, stratified by age					
	**Total (N= 1014)**	**0-5 years (N= 48)**	**6-11 years (N= 268)**	**12-17 years (N= 698)**	**P-value**
ED presentation, n (%)	120 (11.8)	8 (16.7)	30 (11.2)	82 (11.7)	0.55
ED triage category, n (%)					0.91
Resuscitation	0	0	0	0	
Emergency	14 (11.7)	<5	4 (13.3)	9 (11.0)	
Urgent	62 (51.7)	3 (37.5)	15 (50.0)	44 (53.7)	
Semi/ non-urgent	44 (36.7)	4 (50.0)	11 (36.7)	29 (35.4)	
ED Length of stay (hours), median (IQR)	2.8 (1.9, 4.1)	2.4 (1.8, 4.0)	3.1 (1.8, 4.0)	2.8 (2.0, 4.2)	0.89
ED discharge destination, n (%)					0.26
Home	82 (68.3)	5 (62.5)	21 (70.0)	56 (68.3)	
Short stay unit	18 (15.0)	2 (25.0)	7 (23.3)	9 (11.0)	
Ward	6 (5.0)	1 (12.5)	0 (0.0)	5 (6.1)	
Other	14 (11.7)	0 (0.0)	2 (6.7)	12 (14.6)	
In-hospital Outcomes					
Admitted	5 (4.2)	<5	0	<5	0.19
Hospital length of stay (days), median (IQR)	2 (2-2)	3 (3-3)	3 (3-3)	2 (2-2)	0.20
ICU admission	0	0	0	0	0
ICU length of stay (hours), median (IQR)	0	0	0	0	0
Final Diagnosis Category, n (%)					0.27
Cardiovascular	5 (4.5)	0 (0.0)	0 (0.0)	5 (6.8)	
Respiratory disease	19 (17.1)	<5	<5	14 (18.9)	
Gastroenterology	<5	0 (0.0)	1 (3.4)	<5	
Mental health	<5	0 (0.0)	<5	<5	
Musculoskeletal	12 (10.8)	0 (0.0)	<5	10 (13.5)	
Non-specific chest pain	45 (40.5)	<5	14 (48.3)	28 (37.8)	
Other specialties	22 (19.8)	<5	8 (27.6)	11 (14.9)	
72-hour outcomes					
Adverse event	0	0	0	0	0
All-cause mortality	0	0	0	0	0
EMS recontact	<5	0	<5	<5	0.13
30-day outcomes					
All-cause mortality	0	0	0	0	0
EMS recontact	16 (13.3)	<5	<5	11 (13.4)	0.5
90-day outcomes					
All-cause mortality	<5	0	0	<5	0.79
EMS recontact	25 (20.8)	<5	<5	19 (23.2)	0.5

Abbreviations: EMS, Emergency Medical Service; ED, Emergency Department; ICU, Intensive Care Unit; IQR, Interquartile range.

© 2025 Okyere D et al. *JAMA Network Open*.
