# Supplementary material for: Incidence, Diagnoses, and Outcomes of Pediatric Nontraumatic Chest Pain Attended by Ambulance
Source: JAMA Netw Open. 2025 Sep 26;8(9):e2533962. doi: 10.1001/jamanetworkopen.2025.33962 (PMC12475941; doi:10.1001/jamanetworkopen.2025.33962)
Supplement: Supplement 2. — Data Sharing Statement [file jamanetwopen-e2533962-s002.pdf]

Data Sharing Statement

Okyere. Incidence, Diagnoses, and Outcomes of Pediatric Nontraumatic Chest Pain Attended by Ambulance. *JAMA Netw Open*. Published September 26, 2025. doi:10.1001/jamanetworkopen.2025.33962

Data
Data available: No

Additional Information
Explanation for why data not available: The data that supports the findings of this study are available from Dr. Nehme upon reasonable request. The data is not publicly available due to ethical and privacy restrictions.
